# Nonsteroidal Anti-Inflammatory Drugs and Prostatic Diseases

**DOI:** 10.1155/2014/436123

**Published:** 2014-05-12

**Authors:** Hitoshi Ishiguro, Takashi Kawahara

**Affiliations:** ^1^Photocatalyst Group, Kanagawa Academy of Science and Technology, 3-25-13 Tonomachi, Kawasaki-ku, Kanagawa, Kawasaki 210-0821, Japan; ^2^Department of Urology, Yokohama City University Graduate School of Medicine, 3-9 Fukuura, Kanazawa-ku, Kanagawa, Yokohama 236-0004, Japan

## Abstract

Prostatic diseases are characterized by increased activity of cytokines, growth factors, and cyclooxygenases- (COX-) 1 and 2. Activation of COX-1 and COX-2 results in increased levels of prostaglandins and the induction of angiogenic, antiapoptotic and inflammatory processes. Inhibition of COX enzymes by members of the widely used nonsteroidal anti-inflammatory drug (NSAID) class of drugs decreases prostaglandin production, and exerts a variety of anti-inflammatory, antipyretic, and antinociceptive effects. While numerous *in vitro*, *in vivo*, and clinical studies have shown that NSAIDs inhibit the risk and progression of prostatic diseases, the relationship between NSAIDs and such diseases remains controversial. Here we review the literature in this area, critically analyzing the benefits and caveats associated with the use of NSAIDs in the treatment of prostatic diseases.

## 1. Introduction

The initiation and progression of prostatic diseases involve a variety of factors, including the amplification and mutation of genes encoding the androgen receptor, tumor suppressor genes, oncogenes, growth factors, and cytokines, in addition to other processes such as infection [1–4]. Given the role of inflammation in the development and progression of prostatic disease, it has been suggested that inhibition of inflammation would decrease the risk of prostatic diseases. Nonsteroidal anti-inflammatory drugs (NSAIDs) are widely used in the world for their antinociceptive, anti-inflammatory, and antipyretic effects in many diseases, including prostate cancer and prostatic disease. In contrast, several studies have reported that the use of NSAIDs* increases* the risk of prostatic diseases and the relationship between this class of drugs and prostatic disease remains controversial. Current opinion suggests that NSAID treatment would be beneficial for most prostatic diseases, in particular benign prostatic hyperplasia (BPH) and prostate cancer. In this review, we discuss the relationship between NSAIDs and prostatic diseases.

## 2. NSAIDs

**Figure 1:**
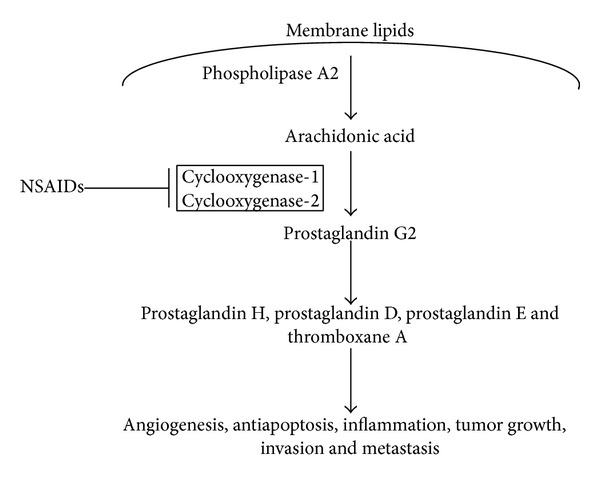
Schematic of the mechanism of action of NSAIDs. NSAID inhibition of cyclooxygenase-1 and/or cyclooxygenase-2 suppresses prostaglandin G2 production, promoting apoptosis and blocking angiogenesis, inflammation, and tumor progression.

The primary mechanism of action of NSAIDs is the inhibition of the activity of cyclooxygenase enzymes (COX-1 and COX-2) and a consequent reduction in prostaglandin levels [5]. COX-1 is constitutively expressed in most tissues and has important roles in tissue homeostasis, particularly in the stomach and kidney, as well as in blood clotting. In contrast, expression of COX-2 is induced by cytokines or growth factors [6]. Both enzymes convert arachidonic acid to prostaglandin G2 (Figure 1), which is in turn converted to various mediators of inflammation, including prostaglandin H, prostaglandin E, prostaglandin D, and thromboxane A.

NSAIDs are classified into two groups: COX-2 nonselective NSAIDs, which inhibit both COX-2 and COX-1 and COX-2 selective NSAIDs. Since COX-1 inhibition has been associated with severe side effects such as gastrointestinal bleeding and damage to gastric mucosa [7], there has been an emphasis on the development of COX-2 selective NSAIDs. COX-2 selective NSAIDs have been shown to inhibit inflammation without damaging the gastric mucosa [8], although some have been linked with cardiovascular toxicity [9].

Given the myriad adverse side effects of classical NSAIDs, increasing attention is being focused on nitric oxide-donating NSAIDs (NO-NSAIDs), which are associated with fewer side effects [10]. NO released from NO-NSAIDs inhibits gastrointestinal bleeding and damage to the gastric mucosa by increasing blood flow and mucus secretion. Moreover, NO-NSAIDs have been shown to be more effective inhibitors of cancer cell growth than classical NSAIDs [10]. Collectively these data suggest that NSAIDs have potential as a novel class of drugs for the prevention of prostatic diseases and prostate cancer.

## 3. Prostatitis

According to the NIH consensus classification of prostatitis syndromes includes 4 categories. These four categories include (1) acute bacterial prostatitis, (2) chronic bacterial prostatitis, (3) chronic prostatitis/CPPS consisting of A: inflammatory and B: noninflammatory, and (4) asymptomatic inflammatory prostatitis [11]. While antibacterial drugs are effective in the treatment of acute bacterial prostatitis, they are less effective in the treatment of the other types of prostatitis. As a consequence, therapy for chronic prostatitis is primarily aimed at managing its symptoms. COX-2 selective NSAIDs have been shown to abrogate or partially relieve dysuric symptoms in 66% and 17% of chronic prostatitis patients, respectively, and to improve inflammatory symptoms in 54% of patients [12]. In a 2003 study comparing the efficacy of different NSAIDs in the treatment of chronic prostatitis [13], a total of 161 chronic prostatitis patients were randomized into three groups treated with 25 mg and 50 mg rofecoxib or placebo, respectively, for 6 weeks. The results indicated that treatment with 50 mg rofecoxib effected a statistical improvement in the quality of life of the patients. Collectively, these data indicate that treatment with NSAIDs might hold many benefits for chronic prostatitis patients.

## 4. Benign Prostate Hyperplasia

Recent* in vitro* and epidemiological evidence has shown that age, genetics, endocrine status, inflammation, and lifestyle are risk factors for BPH and/or lower urinary tract symptoms (LUTS) [14]. Inflammation has been linked with the development and progression of BPH [15, 16], and several studies have reported the presence of intraprostatic inflammatory infiltration in BPH tissues [17, 18]. The inflammatory cytokine IL-17, which is not expressed in normal prostate, has been shown to be expressed in inflammatory prostate [19]. Moreover, COX-1 and COX-2 are expressed in BPH tissues [20–23], and elevated COX-2 expression has been associated with increased levels of Bcl-2 and cell proliferation in BPH [23]. Given the inhibitory effect on COX-1 and COX-2 activity by NSAID treatment, would be anticipated to reduce the risk of BPH development and progression.

In a 2005 study [24], 46 patients with LUTS and BPH were divided to two groups and treated with finasteride only or finasteride + rofecoxib, respectively, for 24 weeks. Compared with the finasteride only group, patients in the finasteride + rofecoxib group had a higher decreasing of average international prostate symptom score (IPSS) after 4 weeks and faster overall relief of BPH symptoms. In another study, compared with LUTS and BPH patients treated with an *α*-blocker alone, patients treated with a COX-2 inhibitor + an *α*-blocker had a higher decreasing of average IPSS, quality of life (QoL) index, and overactive bladder symptoms score (OABSS) [25]. Jhang et al. showed that an *α*-blocker combined with celecoxib group decreased the IPSS score, but not significantly, compared to *α*-blocker monotherapy group [26]. Moreover, nocturia associated with LUTS has been shown to be improved by treatment with sedatives and analgesics, including COX-2 inhibitors [27].

Daily use of NSAIDs has been shown to result in improved urinary symptoms, increased urine flow rate, and decreased prostate volume and prostate-specific antigen levels [28]. In addition, administration of NSAIDs reportedly decreased both IPSS and mean nocturnal urination frequency, another well-known symptom of BPH, suggesting that NSAIDs are a novel option to improve symptoms of BPH [29]. While some studies have reported a lack of side effects associated with NSAID treatment [30], others have reported the opposite results. For example, in a cohort study of NSAID and risk of BPH, using 4,735 men without BPH as a baseline, Schenk et al. found that NSAIDs were not associated with the risk of BPH [31]. In contrast, other studies have reported that NSAIDs increased the risk of BPH [32, 33]. Moreover, acute urinary retention has been associated with the use of COX-2 selective NSAIDs but not with the use of nonselective NSAIDs [34].

While data are limited, several* in vitro* and* in vivo* studies have probed the mechanism(s) underlying the improvement in BPH and its symptoms effected by NSAIDs. Ibuprofen and aspirin, two commonly used NSAIDs, have been shown to decrease viability and suppress proliferation of BPH cell lines [35]. Moreover, certain animal models of BPH are characterized by elevated expression of the enzymes COX-2 and 5-lipoxygenase (5-LOX) that also regulates inflammation [36]. Treatment of these animals with a dual inhibitor of COX and 5-LOX decreased prostaglandin E2 levels and expression of the antiapoptotic factor Bcl-2 and increased expression of the proapoptotic factors Bax and caspase-9, resulting in the induction of apoptosis.

In summary, while the determining of the efficacy of NSAIDs in the treatment of BPH requires further clinical studies,* in vitro* evidence suggests that NSAIDs might be beneficial for alleviating symptoms in BPH patients and for reducing the risk of the development or progression of the disease in unaffected individuals.

## 5. Prostate Cancer

Inflammation arising from a variety of physiological insults is thought to be a major cause and promoter of various cancers, including prostate cancer [37–40]. Inflammation of the prostate is associated with the induction of cytokines, chemokines, and growth factors, as well as COX-2, which is also overexpressed in prostate cancer [21, 41]. Given their anti-inflammatory roles in the reduction of COX activity and prostaglandin synthesis, therefore [37, 42], it has been speculated that treatment with NSAIDs might reduce the risk of prostate cancer [43].

*In vitro* studies have provided evidence pointing to the suppression of prostate cancer development and progression by NSAIDs. For example, celecoxib, a COX-2 selective NSAID, has been shown to induce apoptosis in PC3 and LNCaP cells via inhibition of Akt and activation of caspase-3 [44, 45] and G1 arrest [46]. The fact that these cell lines do not express COX-2 indicates that celecoxib inhibits prostate cancer cell line growth via a COX-2 independent mechanism. In support of this notion, a celecoxib analog deficient in COX-2 inhibition has been shown to inhibit prostate cancer cell growth, also via repression of Akt and G1 arrest [46].

Other studies have investigated the relationship between NSAIDs, androgen signaling, and radiation therapy in the context of prostate cancer. Compared with parental LNCaP cells, LNCaP cells expressing COX-2 are more resistant to radiation therapy, and treatment with the COX-2 inhibitor diclofenac enhanced the effects of radiation therapy on these cells [47]. Similar results were also obtained in xenograft models [47]. NSAIDs have also been shown to delay the progression of prostate tumors from androgen-dependent to androgen-independent growth [48]. NSAIDs delayed the regrowth of LNCaP xenografts in castrated SCID mice. Another paper showed the possibility for the combination of hormone ablation therapy and NSAID treatment [49]. Celecoxib treatment increases the efficacy of androgen ablation therapy.

Studies of the transgenic adenocarcinoma of the mouse prostate (TRAMP) model, which has elevated COX-2 expression and activity, further suggest the potential of NSAIDs in retarding prostate cancer development and progression. In this model, celecoxib suppresses expression of androgen receptor, COX-2, NF*κ*B, and vascular endothelial growth factor (VEGF), reduces prostaglandin E2 levels, and increases levels of E-cadherin, *α*-catenin, and *β*-catenin [50, 51]. In the same model, celecoxib inhibits the development of prostatic intraepithelial neoplasia and adenocarcinoma in the prostate in a dose-dependent manner [52, 53]. The net effect of celecoxib in this model is to suppress invasion and metastasis, induce apoptosis, and enhance overall survival. NSAIDs have also been shown to increase cell cycle arrest and apoptosis via regulation of cell cycle regulatory protein in an animal model of chemical induction of prostate cancer [53]. Collectively these experimental reports suggest that NSAIDs may be beneficial for the treatment of advanced prostate cancer.

By virtue of their decreased side effects, NO-NSAIDs are gaining attention as an alternative to classical NO-NSAIDs side effects. NO-NSAIDs have been shown to inhibit cancer cell growth [10] and, in prostate cancer cells specifically, have proapoptotic and anti-invasive properties which exceed those of classical NSAIDs [54–57]. Moreover, NO-NSAIDs directly inhibit hypoxia-inducible factor-1*α* (HIF-1*α*), a transcriptional activator of VEGF, and other cancer-related genes [58]. Based on these and other studies, NO-NSAIDs show considerable potential as an alternative to classical NSAIDs for the treatment of prostate cancer.

Although* in vitro* studies point to the ability of NSAIDs to protect against cancer progression, results from clinical studies are more inconsistent. Many studies have linked NSAIDs with a reduced risk of prostate cancer [59–64]. For example, aspirin and other NSAIDs have been shown to decrease plasma prostate serum antigen (PSA) levels and to reduce the risk of prostate cancer (OR 0.82, 95% CI 0.68–0.99). Daily use of aspirin also reduced further prostate cancer risk. Smith et al. investigated the effect of NSAIDs on serum PSA after radical prostatectomy or radiation therapy in 78 men randomly divided into placebo (40 men) and celecoxib (38 men) groups. Relative to the placebo group, individuals in the celecoxib group had a 2-fold higher PSA doubling time and significantly decreased PSA velocity [61]. Moreover, combination treatment with an *α*1-blocker and COX-2 inhibitor has been shown to decrease serum PSA in 53.3% of patients [26]. In addition, administration of 200 mg celecoxib twice daily to 20 patients who had undergone radical prostatectomy or radiation therapy effected a reduction in serum PSA in 60% of individuals [65]. In further support of these findings, daily consumption of more than six aspirins has been shown to reduce prostate cancer risk (OR 0.76, 95% CI 0.60–0.98) [60]. In contrast, other studies have failed to associate aspirin with a reduced risk of prostate cancer [66, 67], and one has suggested that treatment with NSAIDs* increases* the risk of prostate cancer (odds ratio (OR) 1.33, 95% confidence interval (95% CI) 1.07 to 1.64) [68]. Interestingly, Leitzmann et al. indicated that, while aspirin reduced the risk of metastatic prostate cancer risk, it did not protect against the risk of prostate cancer [69]. Based on these observations, the association between NSAIDs and prostate cancer risk requires further study.

## 6. Conclusions

Many studies have demonstrated a link between inflammation and prostatic diseases, such as BPH and prostate cancer. Although there are some suggestions that NSAIDs increase the risk of prostatic diseases, most of studies suggest that NSAIDs have potential to improve symptoms in, and reduce the risk of, prostatic diseases.
